# Evaluation of PCA3 and multiparametric MRI’s: collective benefits before deciding initial prostate biopsy for patients with PSA level between 3-10ng/mL

**DOI:** 10.1590/S1677-5538.IBJU.2015.0155

**Published:** 2016

**Authors:** Sezgin Okcelik, Hasan Soydan, Ferhat Ates, Ufuk Berber, Hasan Saygin, Güner Sönmez, Kenan Karademir

**Affiliations:** 1 Department of Urology, Beytepe Military Hospital, Ankara, Turkey;; 2 Department of Urology Haydarpasa Training Hospital, Istanbul, Turkey;; 3 Department of Pathology, Corlu Military Hospital, Tekirdag, Turkey;; 4 Department of Radiology, Haydarpasa Training Hospital, Istanbul, Turkey

**Keywords:** prostate cancer antigen 3, human [Supplementary Concept], Prostate, Magnetic Resonance Imaging

## Abstract

**Objective:**

To analyze the contribution of multiparametric MRI and PCA3 assay, pre- decision of initial biopsy in PSA level between 3-10 ng/mL patients with normal digital rectal examination(DRE).

**Materials and Methods:**

PSA level 3-10 ng/mL ,patients, with normal DRE results and no previous prostate biopsy history, were included in this study. Each patient underwent multiparametric MRI one week before biopsy. Urine sample taking for PCA3 examination preceded the biopsy. Systematic and targeted biopsies were conducted. Patients with high PSA levels were seperated into two groups as: high PCA3 scored and low PCA3 scored. Then each group was divided into two sub-groups as: MRI lesion positive and negative. Tumor incidence, positive predictive values(PPV) and negative predictive values(NPV) were calculated.

**Results:**

53 patients were included between February 2013 and March 2014.Mean age 61.22 ± 1.06. Mean PSA value 5.13 ± 0.19 ng / mL. Mean PCA3 score 98.01 ± 23.13 and mean prostate size was 48.96 ± 2.67 grams. Fourty nine patients had both PCA3 score and multiparametric MRI. The PCA3’s PPV value was 58.33%. If multiparametric MRI lesions are added to high PCA3 scores , the PPV appears to elevate to 91.66%. NPV of PCA3 was 96%. NPV was 95% when there was no lesion in the multiparametric MRI with low PCA3 scores. Sensitivity was 91.66% , specificity was 95% respectively.

**Conclusion:**

Adding multimetric MRI can also support biopsy decision for patients with high PCA3 value. When PCA3 value is low, patients can be survailled without any need to take a MRI.

## INTRODUCTION

Prostate cancer (CaP) is the most commonly diagnosed cancer in men (1). CaP diagnosis relies on prostate specific antigen (PSA), digital rectal examination (DRE) and transrectal ultrasonography (TRUS). However, prostate is the only solid organ on which biopsy is made without seeing the lesion. Thirty per cent of tumors can be missed with TRUS-guided biopsies (2). As some tumors are missed, some clinically insignificant tumors are also extra detected. To avoid these occurrences and to increase the success rate, MRI is performed prior to the second biopsies and MRI guided biopsies are done according to lesions. Today, MRI evaluation is done before initial biopsies to increase this success (3). Although several MRI methods are used, the most sensitive and specific MRI method is multiparametric MRI which is a combination of several MRI methods (4). PCA3 is a non-coding mRNA which is isolated from initial urine after prostate massage (5). In studies after the identification for the first time in 1999, it has been shown that PCA3 is superior to PSA for the presence of prostate cancer (6). Subsequently, it has been used used prior to the initial biopsy (7).

In our study, we investigated the collective benefits of PCA3 and multiparametric MRI for grey area patients whose DRE are normal and serum PSA values are between 3-10ng/mL before initial biopsy decision.

## MATERIALS AND METHODS

The study was planned as a prospective and single-centered. Consent of the local ethics committee for this study was taken with B.30.2.IST.0.30.90.00/16077 numbered decree of Dean of the Cerrahpasa Faculty of Medicine Clinical Research Ethics Committee in 8 June 2012. Serum PSA level 3-10ng/mL patients with normal digital rectal examination scheduled for initial prostate biopsy were included in the study between February 2013 and March 2014. Each patient underwent multiparametric MRI a week before biopsy. After 3-minute prostate massage 20-30mL initial urine sample for PCA3 examination were taken pre-biopsy.

Although biopsy was performed schematically, additional cores were taken from lesions shown in the multiparametric MRI.

## MRI STUDY

Each patient underwent multiparametric MRI evaluation one week before biopsy. Siemens Avanto 1.5 Tesla MRI equipment was used. T2 sequence MRI, diffusion MRI, dynamic contrast-enhanced MRI and MRI spectroscopy were evaluated in multiparametric MRI. Intensity loss regions in T2 sequence MRI, diffusion loss zones in diffusion MRI, contrast involvement and after the early wash-out common areas in dynamic contrast-enhanced MRI, (choline+creatine)/citrate>3SD (this ratio is above 0.86) regions in spectroscopic MRI were considered to be significant. Biopsies were directed to these regions, described and accepted as significant by at least two MRI methods and multiparametric MRI.

## PCA3 STUDY

Initial 20-30cc urine sample after 3 minutes prostatic massage was taken from each patient before the biopsy. Massage from lateral to medial and from baseline through the apex was conducted. After urine was centrifuged within 15 minutes, supernatant section was discarded and RNA preservative was added for later work. The mix was stored at-80ºC. PCA3 study was done with real time multiplex PCR posteriorly (PCA3 mRNA copy number/PSA mRNA copy number) X 1000 formula was used for PCA3 score calculation (5).

**Figure 1:**
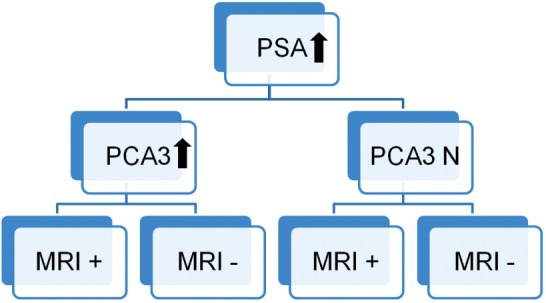
Study Diagram.

Patients with high PSA levels were divided into two groups: with high PCA3 score and low PCA3 score. Then each group was divided into two groups: with MRI lesion positive and negative. Tumor incidence and positive predictive values for patients with high PSA, high PCA3 score and positive multiparametric MRI were calculated. Tumor incidence and negative predictive values in patients with high PSA level and low PCA3 scores and negative multiparametric MRI lesions were calculated as well (Figure-1).

## RESULTS

Fifty three patients were included in our survey conducted between February 2013 and March 2014. One patient’s only urine was sent for PCA3 study without MRI screening. Two patient’s urine wasn’t examined for PCA3, because the urine wasn’t taken to the laboratory within 15 minutes and urine PCA3 RNA preservative could not be added. For one patient’s urine sample didn’t work in PCR, and it was disregarded for PCA3 study.

The mean age of patients was 61.22±1.06. The mean PSA value was 5.13±0.19ng/mL. The mean PCA3 score was 98.01±23.13 and the mean prostate size was 48.96±2.67grams. Digital rectal examination did not reveal any abnormalities in any patient (Table-1).

**Table 1:** General Features.

	Age	ProstateVolume	PSA	PCA3
Mean	61.22	48.96	5.13	98.01
St Deviation	1.06	2.67	0.19	23.13
Median	62	45	5	37.28
St Deviation	7.73	19.47	1.45	163.6
Minimum	43	17	3	0
Maximum	79	93	8.9	1000

Mean biopsy cores were 12.73±0.13. Prostate cancer was detected in 19 of 53 (35.8%) patients. Tumor detected mean cores was 3.21±0.45. ASAP was found in two patients, Gleason 3+3 PCa in thirteen patients and Gleason 3+4 PCa in four patients. None of the patients had Gleason 4+3 or above tumor grade. Two patients’ second biopsy conducted after two months was reported as benign, whose first biopsies were ASAP. As the second biopsies were benign, these two patient’s results were accepted as benign for further evaluation.

Fifty patient’s urine samples were suitable for PCA3 evaluation. Prostate cancer was detected in 15 of them. PCA3 scores were over 35 in 25 patients. In 14 (56%) of these 25 patients tumor was detected. PCA3 score was low in 25 patients. In 1 (4%) of these 25 patients, tumor was detected. Tumor of this patient was present in 1 core and Gleason score was 3+3.

In patients with high PCA3 score tumor detection rate was high and statistically significant (p=0.0001) (Table-2).

**Table 2:** Tumor Detection According to PCA3 Score.

		Tumor Negative	Tumor Positive		P
**PCA3**	<35	24	1	25	0.0001
	>35	11	14	25	

In our survey PCA3’s sensitivity was 93.33%, specificity 68.57% for the threshold of PCA3 of 35. PCA3’ s positive predictive value was 56%, and negative predictive value was 96%. PCA3 score was not related to prostate volume and patient’s age (Table-3).

**Table 3:** Relationship Between PCA3 and Age and Prostate Volume.

		R	P
**PCA3**	Age	0.002	0.988
	Prostate volume	-0.018	0.904

PCA3 score was not significantly different between Gleason score 3+3 and 3+4 tumors (Table-4).

**Table 4:** Relationship Between PCA3 and Gleason Score.

	Biopsy Gleason	Count	Mean	Standard Deviation	p
**PCA3**	6	12	1.91	0.28	0.635
	7	3	2	0	

Multiparametric MRI lesions were detected in 18 patients (when seen at least in two MRI they were accepted as multiparametric MRI lesion). Twelve tumors were screened in 18 lesions (66.6%). Twenty nine lesion negative patients among 34 had no tumor (85.2%).

Significantly more tumors were found in the MRI lesion positive patients than MRI lesion negative patients (p=0.0001) (Table-5). Sensitivity of multiparametric MRI was 70.58% and specificity was 82.80%. Positive predictive values were calculated as 66.60% and negative predictive values were 85.2%.

**Table 5:** Comparison of Multiparametric MRI and Biopsy.

		Multiparametric MRI Lesion Tumor-	Multiparametric MRI Lesion Tumor+	Total	p
Multiparametric MRI Lesion	**Lesion-**	**29**	**5**	**34**	0.0001
	**Lesion+**	6	12	18	

**Total**		**35**	**17**	**52**	

Patients with both PCA3 score and multiparametric MRI available totaled 49. There were 24 patients with high PCA3 scores. Fourteen of these patients had tumors. Accordingly, the PCA3’s positive predictive value detected was 58.33%. Twelve patients had both high PCA3 score and multiparametric MRI lesion and tumor was detected in 11 of these. Our study revealed that if multiparametric MRI lesions are added to high PCA3 scores, the positive predictive value appears to increase to 91.66%.

**Figure 2:**
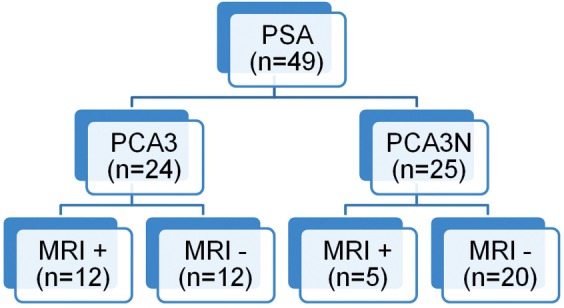
Diagram of the patients with high PSA level, have PCA3 value and multiparametric MRI.

There were 25 patients with normal PCA3 scores and only one patient had tumor whereas 24 patients didn’t. Accordingly, the negative predictive value of PCA3 was calculated as 96%. In 20 of these 25 patients, any MRI lesion was observed. Of these patients, tumor was detected in only one patient. Negative predictive value was calculated as 95% when no lesion existed in the multiparametric MRI with low PCA3 scores. Sensitivity was 91.66% and specificity was 95% respectively (Figure-2).

## DISCUSSION

The systematic TRUS-guided prostate biopsy still continue to be the standard method when cancer is suspected (8). However, prostate biopsy is a procedure where can occur serious complications such as urinary infections that can go to sepsis (9), rectal bleeding (10), catheterization requirement for retention (10). Also pre-biopsy anxiety affects the patients psychologically (11). On the other hand, decreasing PSA threshold when deciding on biopsy can increase the rates of clinically insignificant tumors (12). Therefore, biopsy decision should be taken more carefully and new diagnostic methods should be developed for the detection of tumors that are required to be treated.

Nowadays PCA3 is usually used to decide on second biopsy in patients with continuous PSA elevation whose first biopsies were reported as benign (13). Considering the pre-biopsy anxiety and post-biopsy complications PCA3 is also used in making decision for the first biopsies (7). It was observed that PCA3 is significantly higher in patients with tumors at the Chevli’s 3073 first biopsy planned patients study. This study also showed that higher PCA3 scores were detected in patients with higher Gleason scores (7).

In our survey PCA3’s sensitivity was 93.33% and specificity was 68.57% for the threshold of PCA3 35. PCA3’ s positive predictive value was 56%, and negative predictive value was 96%. In the literature, it’s stated that sensitivity was 47-69%, and specificity ranged from 72 to 79% (14, 15). In our study sensitivity was higher than literature, although specifity was slightly lower, but similar. Positive predictive value of our study was similar to 55% that Leyten and colleagues reported (16). PCA3 in this study performed better than in other studies.

Also PCA3 wasn’t related with age and prostate volume (p=0.988, p=0.904). This can represent that PCA3 is superior to PSA. In our study, detected tumor’s Gleason scores were 6 and 7. No difference was detected between the Gleason scores for PCA3 (p=0.823, p=0.635). There was also no difference between the high grade tumors and low grade tumors for PCA3 in Leyten and his colleague’s study (16). Unlike our study, Busetto and colleagues showed that patients with high-grade tumor had the higher PCA3 scores (13). In our study, a meaningful result could not be obtained because of the low-risk group of patients evaluated and patients with Gleason score 6 and 7 were in close proximity to clinical data.

MRI can be used in several areas of prostate cancer diagnosis and treatment. MRI can be used in patients with ongoing high PSA whose previous biopsy resulted benign, before the second biopsy, to increase the success, to preoperative evaluation of the tumor relationship neurovascular bundles, radiotherapy planning, in focal treatment planning, and for patients undergoing active surveillance (17).

In addition to these areas, today MRI is used before the initial biopsy decision to increase the biopsy success. Although various MRI methods are used, the most sensitive and specific method is multiparametric MRI which is a combination of several multiparametric MRI methods (4). In our study, significantly more tumor was determined in patients with multiparametric MRI lesion compared to patients without multiparametric MRI lesion (p=0.0001). Multiparametric MRI’s sensitivity was 70.58%, specificity was 82.80%, positive predictive value was 66.60%, and negative predictive value was 85.2%. Multiparametric MRI was found to be most useful for detecting tumor in terms of the presence of multiparametric MRI lesions. Although our findings in MRI were very little low compared to literature, they were similar to it. In the literature, multiparametric MRI’s sensitivity was 75%, and specificity was 94% for 0.2cc to larger lesions (18).

There is no study which determines combined benefits of PCA3 score and multiparametric MRI in this patient group in the literature. Fourty nine patients had both PCA3 score and multiparametric MRI available. There were 24 patients with high PCA3 score and fourteen of these patients (58.3%) had tumors. Twelve patients had both high PCA3 score and multiparametric MRI lesion. Tumor was detected in 11 of 12 patients. Our study showed that if multiparametric MRI lesions are added to high PCA3 scores, the positive predictive value appears to increase to 91.66%. There were 25 patients with a normal PCA3 score. Of these 25 patients, only one patient had tumor. Accordingly, the negative predictive value of PCA3 was calculated as 96%. Fradet and colleagues stated that PCA3 positive predictive value was 75% and negative predictive value 84% (19). In Tinzl and colleague’s study positive predictive value was 67% and negative predictive value was 87% (20). Hessels et al. found the negative predictive value of 90% in their evaluation by PCR (21). In our study, the negative predictive value was higher than in literature. Because MRI lesions were low in patients with low PCA3 score, systemic biopsies were taken in this patient group. Some tumors could have been missed in this patient group. In conclusion, the importance of pre-biopsy MRI evaluation for targeted biopsy is emerging.

No lesion was observed in MRI of 20 of 25 patients with normal PCA3 score. Among these patients, tumor was detected only in one patient. Negative predictive value was calculated 95% (no lesion in multiparametric MRI with low PCA3 scores). Sensitivity was 91.66% and specificity 95%.

Our study revealed that using only PSA score for tumor detection, accuracy rate is 35.9%, but this rate raised to 58.33% by adding PCA3. PCA3 score, along with multiparametric MRI findings, elevated this rate to 91.66%. Likewise, in this study we observed that NPV was calculated as 96% using the PCA3 score (<35). There wasn’t any increase in the NPV when we added absence of lesions in the multiparametric MRI. Thus, our data suggest that when patient’s PCA3 is low (<35), we can affirm there is no tumor with 96% of accuracy without MRI study.

## CONCLUSIONS

In patients with PSA level between 3-10ng/mL and normal DRE, PCA3 score and multiparametric MRI seems to provide additional contributions to first biopsy decision for the detection of prostate cancer. When these methods and approaches are combined used, PPV is significantly increased for predicting tumor presence.
